# Social interactions of juvenile rabbits (*Oryctolagus cuniculus*) and their potential role in lagovirus transmission

**DOI:** 10.1371/journal.pone.0271272

**Published:** 2022-07-28

**Authors:** Emma Sawyers, Tarnya E. Cox, Peter J. S. Fleming, Luke K. P. Leung, Stephen Morris

**Affiliations:** 1 Vertebrate Pest Research Unit, New South Wales Department of Primary Industries, Orange, New South Wales, Australia; 2 School of Agriculture and Food Sciences, University of Queensland, Gatton, Queensland, Australia; 3 School of Environmental and Rural Science, University of New England, Armidale, New South Wales, Australia; 4 Fisheries Research, New South Wales Department of Primary Industries, Wollongbar, New South Wales, Australia; University of Oklahoma Norman Campus: The University of Oklahoma, UNITED STATES

## Abstract

Rabbit Haemorrhagic Disease Virus (RHDV), which is a calicivirus, is used as a biocontrol agent to suppress European wild rabbit populations in Australia. The transmission of RHDV can be influenced by social interactions of rabbits; however, there is a paucity of this knowledge about juvenile rabbits and the roles they may play in the transmission of RHDV. We aimed to quantify the social interactions of juvenile (< 900 g) and adult (> 1200 g) rabbits in a locally abundant population in the Central Tablelands of New South Wales, Australia. Twenty-six juvenile and 16 adult rabbits were fitted with VHF proximity loggers to monitor intra- and inter-group pairings. Use of multiple warrens by these rabbits was investigated using VHF base stations at nine warrens and on foot with a hand-held Yagi antenna. Juvenile rabbits were strongly interconnected with both juveniles and adults within and outside their warren of capture, and almost all juveniles were well-connected to other individuals within their own social group. Inter-group pairings were infrequent and fleeting between adults. Both juvenile and adult rabbits used multiple warrens. However, visits to warrens outside their warren of capture, particularly those within 50 m, were more common and longer in duration in juveniles than in adults. The high connectivity of juveniles within and between warrens in close proximity increases potential pathogen exchange between warrens. Therefore, juvenile rabbits could be of greater importance in lagovirus transmission than adult rabbits. The strength of juvenile rabbit inter- and intra-group pairings, and their tendency to use multiple warrens, highlight their potential to act as ‘superspreaders’ of both infection and immunity for lagoviruses and other pathogens with similar lifecycles. Confirmation of this potential is required through examination of disease progress and rabbit age-related immune responses during outbreaks.

## Introduction

Although the European wild rabbit (*Oryctolagus cuniculus*) is a keystone species and recognised as endangered in its native range [1], in Australasia it is a serious introduced pest and subject to suppressive management [2]. The introduction of biological control agents has had profound success in the management of wild rabbits in Australia [3,4]. Myxoma virus was released in Australia in 1950 to control pest rabbit populations [5], which was followed by the introduction of two insects to aid in its transmission [6–8]. However, natural selection led to attenuation of myxoma virus in the field and developing resistance to the disease, both of which resulted in lower mortality and efficacy of population control [9]. The natural outbreak of a new disease in European rabbits in China, Rabbit Haemorrhagic Disease (RHD), in 1984 provided an opportunity for a new biocontrol agent in Australia [10].

Rabbit Haemorrhagic Disease Virus (RHDV) strains cause RHD in rabbits, and all strains are lagoviruses of the Caliciviridae family [10]. The transmission routes of lagoviruses include direct contact through oral, nasal and parenteral sites, and indirectly via faecal shedding, fomites, insect vectors and from predation and scavenging on infected carcasses [5,10–13]. RHDVs have a 1–2 day incubation period before usually killing the susceptible host within 12–36 hours (after onset of clinical signs) [11]. RHDV’s can persist up to three weeks in a carcass above ground [10], or up to three months in carcasses inside warrens [14].

In response to the declining efficacy of myxoma virus in Australian rabbits, two variants of the biological control agent Rabbit Haemorrhagic Disease Virus 1 (RHDV1) were released nationally: the GI.1c variant in 1996 (originally detected in Czechoslovakia); and the K5 variant (G1.1a, originally detected in Korea) in 2017 [4,15–17]. In addition, an exotic strain (GI.4eP-GI.1a, originally detected in China), a new wild strain (GI.1bP-GI.2, originally detected in France) and an endemic non-pathogenic RHDV (RHDV GI.4c) are also present in Australian rabbit populations [18–21]. The non-pathogenic RHDV GI.4c is most prevalent in juvenile (< 900 g) rabbits and offers them partial tempory protection to infection from GI.1c for up to 10 weeks [22].

The presence of the interacting variants complicates the epidemiology of RHD in Australia and the role that juvenile rabbits play in the transmission of RHDV and RHDV GI.4c is somewhat uncertain [20,23,24]. They are potentially important because juveniles can carry and shed both pathogenic and non-pathogenic RHDV [25]. Virus shedding has been detected in juveniles up to 10 weeks after initial infection and they can transmit lagoviruses to female adult rabbits (> 1200 g) from two weeks of age [25]. Juveniles less than 3 weeks of age may be fully immune to lagoviruses and likely do not support replication [8]. They are generally naturally resistant to RHDV1 infection up to six weeks of age [26], can develop immunity to RHDV1 until approximately 10 weeks of age [25], and can be protected from infection of RHDV1 by maternal antibodies up to 12 weeks of age [27]. Transmission of GI.2 by juvenile rabbits creates further uncertainty, as juvenile rabbits may become infected and succumb to the disease with this strain [28]. Juvenile rabbits can also be infected with and carry the endemic non-pathogenic lagovirus (RHDV GI.4c), which provides temporary additional protection and can be transmitted to other susceptible rabbits seven days after infection [22]. If juveniles are infected with RHDV1 during 6–12 weeks of age, immunity may become permanent and provide cross-immunity between lagovirus strains, enabling the establishment of a resistant breeding generation [25,27]. These immunities have implications for the spread and establishment of lagoviruses in wild European rabbit populations and hence the effectiveness of circulating and new lagovirus strains for population biocontrol and the timing of control programs [24]. Because juvenile rabbits can carry pathogenic viruses without developing disease, they are at increased likelihood of playing an important role in influencing the effects of lagoviruses on rabbit populations through their movements and pairings with other rabbits.

Contacts between susceptible and infected animals are key drivers in pathogen transmission [29,30]. Social factors (as described in social networks) influence contact rates and hence the transmission of pathogens (as modelled by disease networks) [31–34], with well-connected individuals having a greater potential to transmit a pathogen to susceptible individuals in other social groups through population mixing [4,33,35]. Lagovirus transmission throughout a population of rabbits depends on social interactions between infected and susceptable individuals, within and between groups, and those interactions are likely to be heterogenous. However, observation and measurement of the close social interactions required to determine direct and indirect pathogen transmission among Australian rabbits has been difficult until relatively recently because of technological limitations. The use of proximity logging devices has advanced knowledge about social network structure and contacts in free-ranging wild animals [36–38]. These devices provide continuous and remote collection of social interaction information including time of day, date, frequency and duration of pairings made between individuals carrying proximity loggers [36,37,39]. These ‘biologgers’ can provide insight into the social interactions of many species [40] that have been difficult to track or observe such as fossorial species (e.g. rabbits [41,42]), gregarious livestock (e.g. cattle [43]) and nocturnally active species (e.g. vampire bats, *Desmodus rotundus*, [44] and raccoons, *Procyon lotor*, [40]).

Recently, proximity loggers and VHF devices have been used to investigate pairing rates (i.e., when a logging device within a given proximity records another logger, which could constitute a contact between the pair of individuals) for pathogen transmission between adult and sub-adult (900 g–1200 g) rabbits [23,33,42], but not between juvenile rabbits and the other age groups. This was mainly due to the difficulty of attaching the logger to a juvenile rabbit [37,44]. Pairing rates of juvenile rabbits and their movements between warrens are required in pathogen transmission modelling to inform the timing of virus release and to predict the efficacy of new strains [45–47]. Although male adult rabbits can travel between warrens to mate [48,49] and females exhibit greater site fidelity [48], adult rabbits are generally unsociable, both within and between warren systems [48]. Juvenile rabbits will remain near the natal warren for about 2–3 weeks following emergence [8,49]. However, they then begin exploring within several hundred meters of their natal warren [32,50] before dispersing to a neighbouring warren [49]. Some juvenile rabbits can travel up to 1.5 km from their natal site during exploration and dispersal events [2]. During exploration, it is likely that more frequent pairings are made with members of surrounding social groups with aggressive and sexual behaviours tending to increase at the time of dispersal [14]. Juvenile rabbits possibly visit multiple warrens and engage in longer and more frequent inter- and intra-group pairings than their adult counterparts. Juvenile rabbits might also be allowed entry to non-natal warrens prior to and during dispersal events when adults are not. If juvenile rabbits enter non-natal warrens, the protective capabilities of maternal antibodies and RHDV GI.4c could allow them to be effective vectors of lagoviruses through infectious contact with the social groups that they visit.

Therefore, juveniles potentially play a more important role in pathogen transmission between warrens than adults and could be the key player in the transmission and persistence of lagoviruses in rabbit populations. Here, we aimed to determine rates of visitation to warren of capture and other warrens by juvenile and adult rabbits, to measure the frequency and duration of inter- and intra-group pairings among adult and juvenile rabbits, and to determine the likely relative importance of juvenile rabbits in lagovirus pathogen transmission between rabbit warrens.

## Methods and materials

### Study site and animals

**Fig 1:**
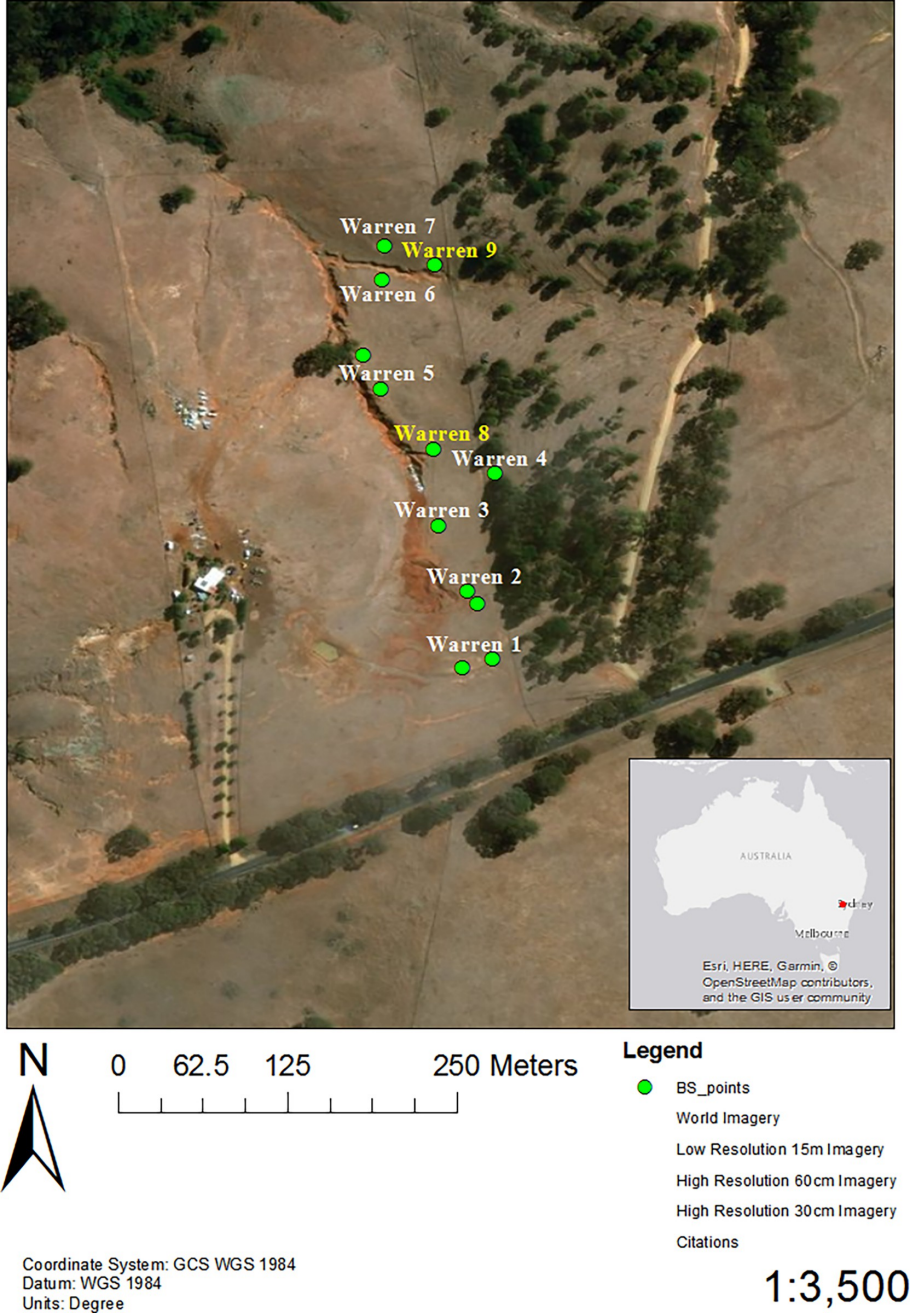
Location of base stations (green circles) in Central West New South Wales, Australia. Trapping of rabbits was conducted on warrens highlighted in white (1–7), while warrens highlighted in yellow were not trapped (8 and 9). Base stations were placed at the centre of warrens (3, 4, 6, 7, 8, and 9) or at either end for larger warrens (warrens 1, 2 and 5), Mapping was undertaken with ESRI software [26].

This study was conducted on a private property located between Orange and Cargo in the Central Tablelands of NSW (-33.367258, 148.881922, elevation 788 m; average annual rainfall 600–1000 mm, Fig 1), during spring–early summer 2017. Rabbit warrens with many burrow openings were centred along a 330-m erosion gully that occupied approximately 5.94 ha of a paddock used for grazing.

Rabbit capture procedures were approved by the Orange Animal Ethics Committee and conducted under animal ethics approval ORA 17/20/004. Ninety-seven treadle-operated, wire-mesh cage-traps (67 x 30 x 30 cm), partially covered with poly shade-cloth, were deployed at burrows and warrens along the gully in the centre of the site (Fig 1). Because adult rabbits are neophobic [2], cages were cable tied open and free-fed weekly with diced carrot for two months prior to trapping. This allowed rabbits to habituate to the traps and carrot bait prior to the capture period.

Trapping was carried out from late September to mid-December when breeding was occurring. Traps were set between 1500 hr and 1700 hr, Monday–Thursday, and checked the following morning at 0600 hr, Tuesday–Friday. Trapping was not conducted during forecasted poor weather conditions (> 10 mm rain, heavy wind, extreme cold). Captured animals were removed from traps and secured in calico bags. All newly captured animals were fitted with a microchip (ID 100/1.4 Trovan Unique Midichip- Microchips, Australia) for identification then weighed. Suitable animals were fitted with proximity loggers (see below). All animals were released at their point of capture. It is possible that the warren of capture (WOC) for some rabbits was not their natal warren or common social group. For this reason, rabbit locations are referred to as their ‘WOC’, rather than ‘natal warren’ [39].

#### Proximity loggers

VHF encounter proximity loggers (Lotek, Havelock, New Zealand,–custom made juvenile model, E2H 162 PID 1607; adult model, E2C 171A) were used to gather information on individual’s social behaviour. In general, each logger emitted a unique ultra-high frequency signal while searching for the frequency of other loggers within a distance pre-set by researchers [36]. When two loggers come within the pre-set distance, a ‘pairing’ on both systems is recorded. Reciprocating logger identification number, date, time and the duration of pairings were recorded and saved to each device’s internal memory. When the devices became separated beyond the set distance and predetermined time interval, we considered the pairing to be concluded.

Prior to deployment, we tested each logger for faults, set the pairing range at 30–50 cm (close proximity deemed to be a pairing between two rabbits), and set the separation time to 30 seconds. Due to concerns about battery life, an additional transmitter (A2455 1.2 g, set at 24 pulses per minute for 140 days- Advanced Telemetry Systems, Isanti, USA) with its frequency matched to the corresponding logger, was glued to the VHF proximity radio-loggers (Sellys® Quick Fix™ super glue). Mesh wings were glued to the ventral side of the logger to increase the surface area for attachment to the rabbits. Given the length of antennas for juvenile loggers (32 cm), antennas were curled to 3 cm diameter, then heat-shrunk into place to limit the chance of entanglement with obstructions in the field.

Loggers were only fitted to juvenile rabbits that were within the 600–750 g weight range (n = 26). This ensured that the data collected would be from rabbits that were still in the juvenile life stage for the duration of the study, but that were large enough for the loggers to be < 5% of their body weight. The hair between the shoulder blades of juvenile rabbits was trimmed and shaved so that the proximity logger could be glued to the animal. Proximity loggers were then secured by folding the surrounding longer guard hairs over the mesh wings [41]. Collars were used to attach VHF proximity loggers to adult rabbits (> 1200 g) [23,42] and all adult loggers were deployed during the study (n = 16). No loggers were attached to sub-adults as this age class was not the focus of this study. All loggers used on juvenile rabbits detached from individuals before they reached 900 g (based on an average growth rate of 10 g per day) due to grooming, hair growth and general wear and tear associated with the fossorial behaviours of the rabbits. The battery life of the logger was ~80 days, allowing for use on multiple juveniles if sufficient battery life remained after detachment. Loggers for adults remained on the same individual for the life of the study. All study animals were humanely killed at the completion of the study to comply with the State’s Wild Rabbits Pest Control Order (*NSW Local Land Services Act*, *2016*), and tissue samples were taken for a wider study investigating the serology of wild European rabbits. To recover loggers and extract the data, adults were either re-trapped and then humanely killed by stunning with a captive bolt followed by cervical dislocation (separating the brain from the spinal cord: Standard Operating Procedure GEN001: Methods of euthanasia [51]), or shot under spotlight by an experienced licenced shooter.

Loggers were fitted to 77% of captured adults and 36% of captured juveniles. The low rate for juveniles was because many were too small. Of the 42 VHF proximity loggers deployed, only two were recovered and re-used after cleaning with 2% hyperchlorite solution. Redeployed loggers were re-labelled with a decimal (e.g. 2.1 and 2.2) to distinguish between the two rabbits. We recovered 40 logging devices at the completion of the study (adult logger numbers 38 and 46 could not be found). All juvenile loggers were found, either on the surface or in warrens. Six loggers (juvenile n = 4, adult n = 2) did not provide data either due to logger failure or no interactions with other logging devices, or provided a series of broken one-second pairings greater than five minutes apart (see Data cleaning below).

#### Base stations

Base stations were used to record pairing information when rabbits come within a predefined distance of an area of interest. We deployed 12 base stations over nine warrens (trapped and un-trapped; Fig 1) to determine visitations by rabbits wearing VHF proximity logging devices. Two base stations were positioned at opposite ends of three larger warrens (1, 2 and 5- Fig 1). Base stations were tested for faults and the detection range set at 1.5 m prior to deployment.

### Data cleaning

#### Proximity loggers

Performance of proximity loggers might not have been identical, which would cause asymmetries in data between paired devices [36]. Orientation and signal obstruction (presence of surrounding objects, size of the animal and fossorial behaviour) may have impacted upon the collection of data [36], and it is important that these limitations are considered.

Using the statistical platform R [52], obscure logger numbers and pairing characters (e.g. alphabetical instead of numeric) were removed from the data. Logger pairings within 12 hours of initial deployment were also removed from the data to reduce the effect of unusual behaviour caused by handling [42].

We considered that each logged one-second proximity was a brief passing encounter that was unlikely to facilitate pathogen transmission because transmission likelihood is a product of contacts and their duration [46]. However, clusters of short sequence pairings of 1-sec between the same individuals would likely be cumulatively sufficient for pathogen transmission. Therefore, to ensure that pairings were of sufficient duration to enable disease propagule transmission between individuals for network analyses, all one-second pairings between the same two individuals within five minutes were conservatively considered sufficient for pathogen transmission. All other one-second pairings were removed from the data (2,050 out of 28,421 encounters).

Two loggers (2.1 and 16) were excluded from this display as they only occurred as ‘encounters’. Blank spaces are used to identify loggers that failed to record data, produced no pairings following data cleaning, recorded invalid pairings or were not recovered at the completion of the study (see text).

#### Base stations

Data from all base stations were cleaned according to rabbit transponder deployment and retrieval dates, as described for VHF proximity loggers. Base station numbers were replaced with warren number, and where two base stations were located on a warren, the data were merged with information on WOC.

#### Field tracking of rabbits

*Rabbits were located using an extended three element folding handheld Yagi antenna and receiver* (Ultra receiver, Lotek, Newmarket, Canada), Monday–Friday at 05:30 and 16:00, and once more at a random time between 22:00 and 02:00 (to eliminate observer bias [53]). This provided additional information on the space rabbits occupied, daytime resting locations and mortalities.

### Analyses

#### Duration of pairings

Total pairing duration was calculated for each logger pair. Using labels *i and j* to denote any pair of loggers, total pairing time with logger *j* was extracted from the logger *i* record and total pairing time with logger *i* was extracted from the logger *j* record. The two totals were never in agreement due to the operational issues described earlier. Therefore, the larger of the two values was taken to represent total time in pairing for the observation period.

**Fig 2:**
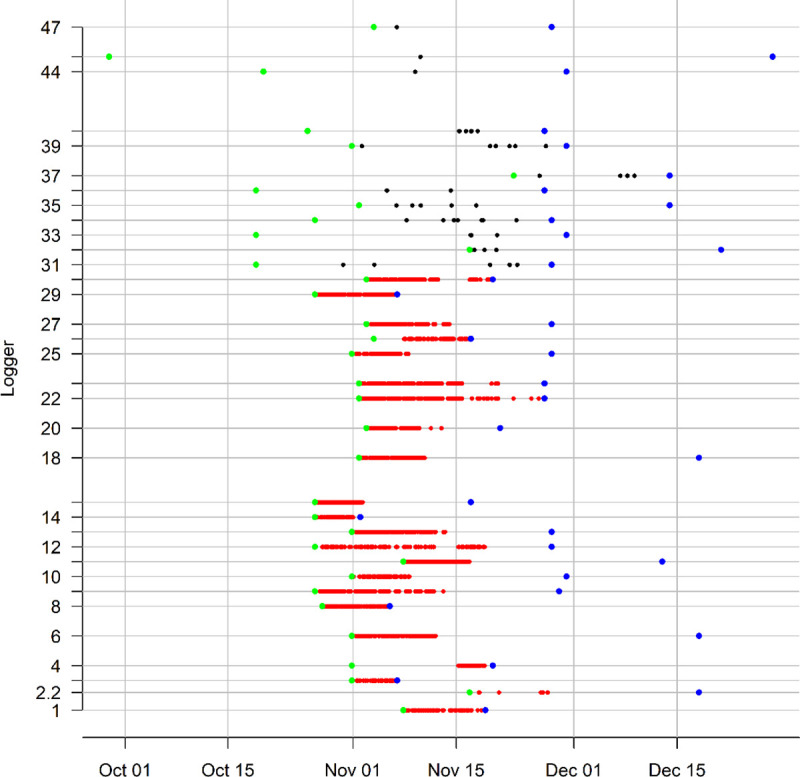
Distribution of total time in pairing for all observed pairs of rabbits classified by age class. Solid symbols and lines denote group averages and 95% confidence intervals.

Pairings were grouped according to age class pairs (i.e. between juvenile rabbits [hereafter ‘J–J’], between adults and juveniles [hereafter ‘A–J’] and between adults [hereafter ‘A–A’]) and the distribution of duration times within each group presented graphically (Fig 2). A formal statistical comparison between pair classes was not conducted due to strong variance heterogeneity and asymmetry within groups even after log_10_ transformation. The log scale durations for each group were summarised by average and 95% confidence intervals (Fig 2) to enable informal statistical comparison.

#### Pairing networks

A network representation of pairing between logger pairs was constructed using the *igraph* package [16] in the R environment [52] by using pair duration to define the graph edges and age class to define the nodes. A similar analysis of interaction between individuals and the base stations was also conducted.

## Results

### Sample population

One hundred individual rabbits were trapped in 297 capture events over 45 trapping nights. Of these rabbits, 76% were juvenile, 22% were adults, and 2% sub-adults were captured. Nine adults were recaptured at least once during trapping events and 64% of the juveniles were trapped twice or more times. Using the total number of individuals that we trapped as a minimum number known alive at the completion of trapping, a minimum density of rabbits across the study site was 16.8 rabbits ha^-1^.

### Pairings between rabbits

After data cleaning, 26,371 pairings were recorded across all individuals (range: 1 sec–1 hr, 29 min and 57 sec). However, most (59.8%) pairings were of 1 sec in duration. There were 10,499 J-J pairings of > 1 sec duration, but only 44 such A-J and 11 such A–A pairings).

Pairings between juveniles were more common and of longer duration than A-J pairings or A-A pairings (Fig 2).

Although there were 630 possible pairings within our sample of 36 rabbits, the total number of observed contact combinations was 79, comprising 56 J–J pairs, 20 A–J and three A–A.

**Fig 3:**
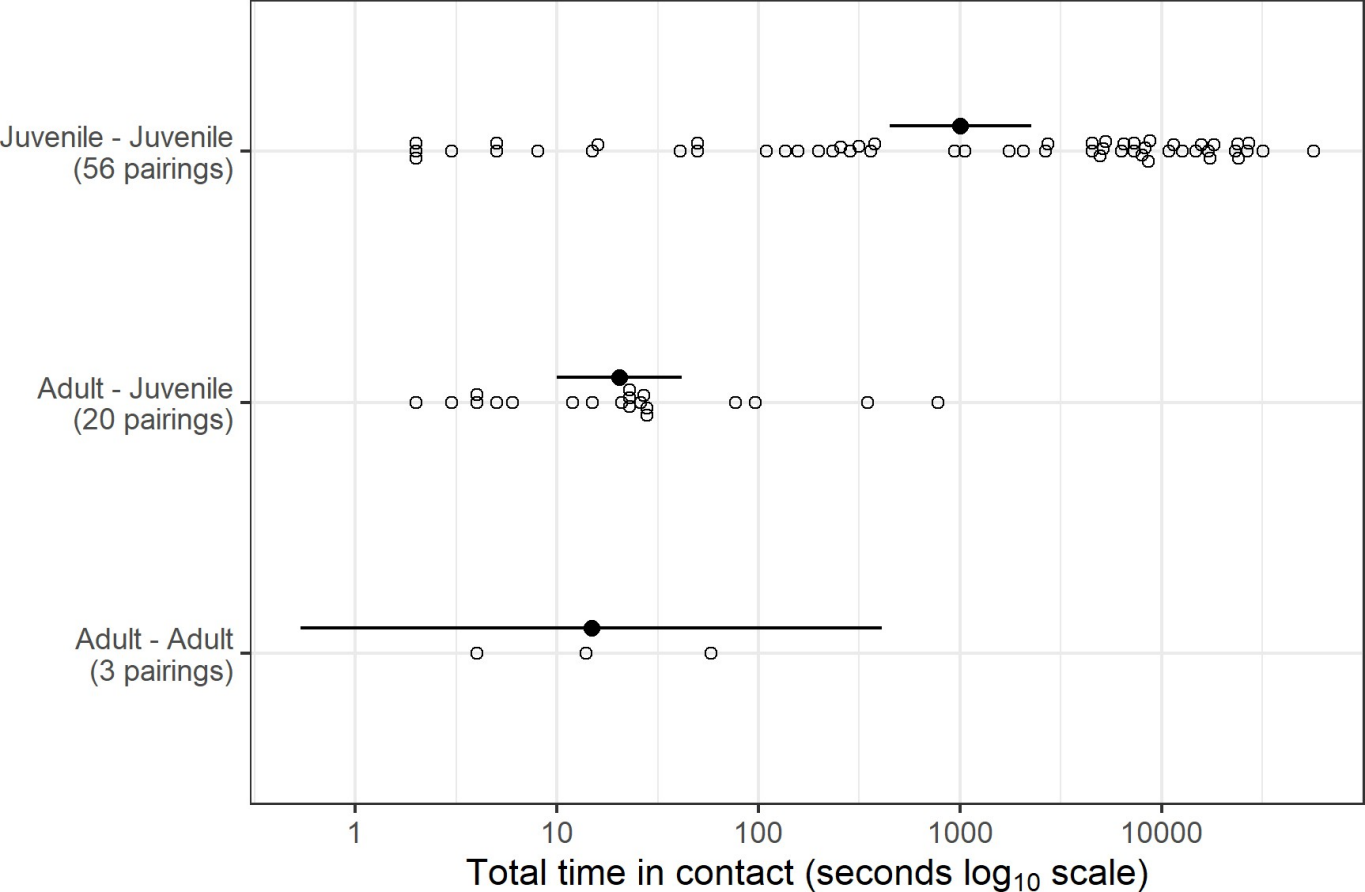
Graphical representation of individual rabbits and pairings between logger pairs (n = 79). Each social group is made up of nodes (circles indicating logger identification number) and connecting lines between nodes. Node colours indicate warren of capture and node size indicates rabbit age class (adult and juvenile). Edge thickness (10 seconds- 5000 seconds) is proportional to total time in pairing (log_10_ scale).

The network analysis indicates a possible eight social pairing groups existed in the study population (Fig 3), as the interactions between rabbits indicated there could have been two social groups coexisting within the one warren system (warren 2, Fig 3). Four of the eight sampled groups consisted of a mix of juvenile and adult rabbits, only adult proximity collars were deployed in warrens 5 and 6 and only juvenile rabbits were sampled in warren 4.

Juvenile rabbits, unlike adult rabbits, made strong connections with individuals from outside of their social group (Fig 3), with more frequent and longer duration pairings with other juveniles from neighbouring warrens. Pairings made between juveniles from warrens 1 and 2, and from 3 and 4, were particularly strong (Table 1). These four warrens were all within 30–50 m of each other (Fig 1). Pairings between juvenile rabbits from more distant warrens occurred (e.g. warren 1 with warren 7), but these J–J pairings were less common (Σ = 378 seconds). Pairing between adults from different social groups were very uncommon (adult intra-group pairings n = 2, Σ = 18 seconds). Inter-group pairings between adults were from warrens 6 and 7, which were less than 10m apart, and may potentially represent one social group.

**Table 1:** Total duration of pairings between pairs of rabbit warrens for three pairs of rabbit age class (J–J = juvenile-juvenile, A–J = adult-juvenile and A–A = adult-adult).

Interaction type	Warren pairs	Total pairing duration (Sec)
**J–J**	1–2	19,986
**J–J**	3–4	2,215
**J–J**	1–7	378
**A–J**	1–2	119
**A–A**	6–7	18
**A–J**	1–7	12
**A–J**	2–6	5
**A–J**	2–7	4
**A–J**	2–3	4
**J–J**	2–2	3
**J–J**	2–4	2

**Fig 4:**
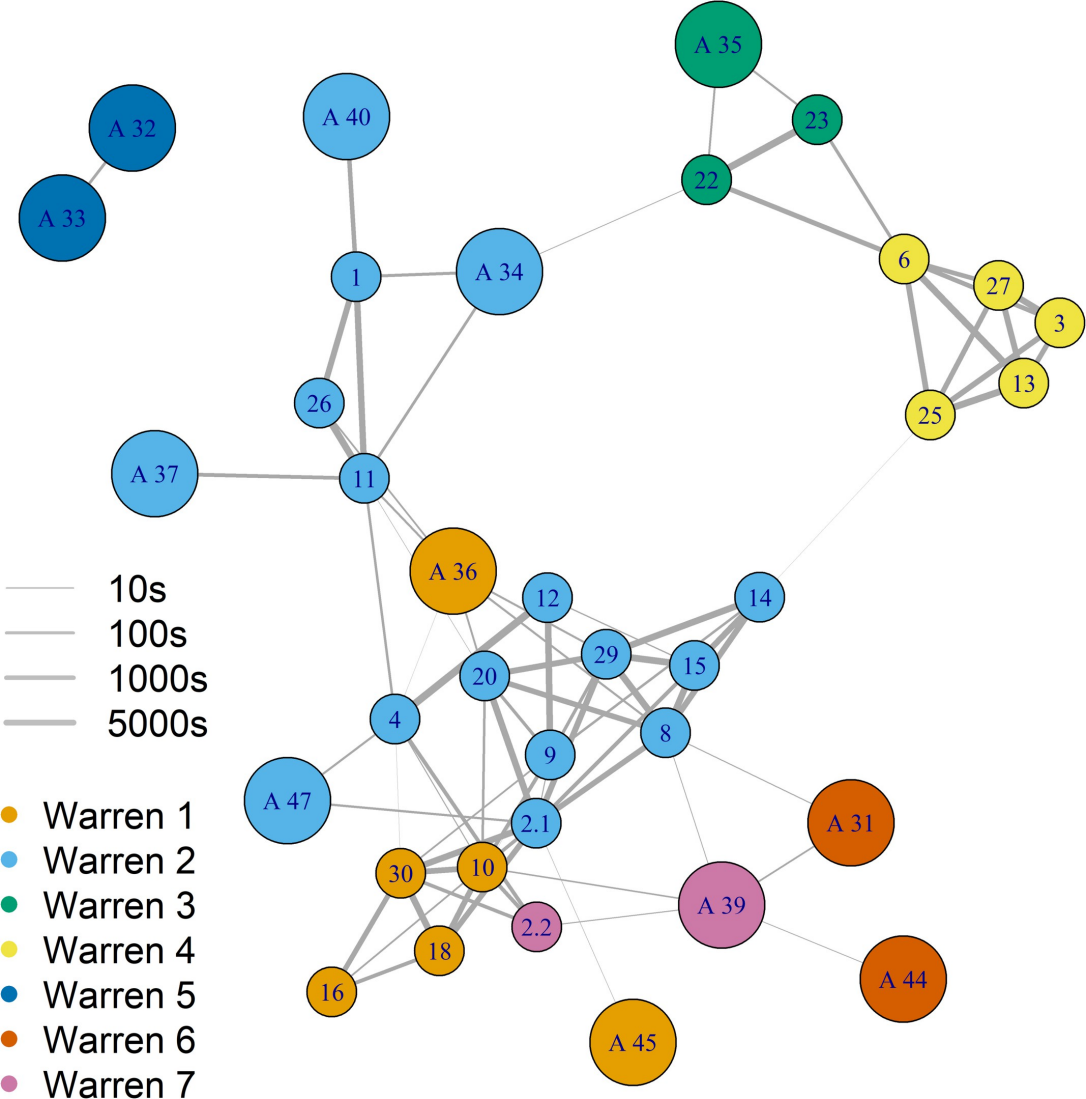
Study time-line showing logger activity (timing and duration of loggers that paired during the study (vertical axis)), between deployment (green points) and retrieval dates (blue points) of juvenile (red) and adult (black) loggers (n = 34 unique loggers with valid pairing data).

Most juveniles from the same social group were connected and shared strong bonds (as indicated by line thickness between nodes: Fig 3). However, some juveniles from the same social group did not pair with each other (9, 4, and 12 with 14 and 8). Juvenile-juvenile connections were stronger than A–J and A–A connections (J–J = 408,491 sec, A–J = 1,406 sec and A–A = 58 sec). In total, juvenile rabbits from within the same social group were connected to more rabbits (juvenile and adults) to each other than the adults of the same social group. For example, adults 37 and 40 were only connected to one juvenile, while almost all juveniles were connected to more than three other rabbits. Adult rabbits were indirectly connected with all individuals within a social group through the high connectivity of their associated juveniles. For example, adult 37 is indirectly connected to juvenile 26 through juvenile 11 and so on. Despite adult rabbits carrying loggers for longer (Fig 4), intra-group connections between adults were infrequent and weak in strength. The longest duration a logger was attached to an adult was 13 weeks, and to juveniles was approximately 3.5 weeks. However, only adult loggers 33 and 32 (58 sec), 31 and 39 (14 sec), and 44 and 39 (4 sec) made pairing during the study period.

## Multiple warren use

**Fig 5:**
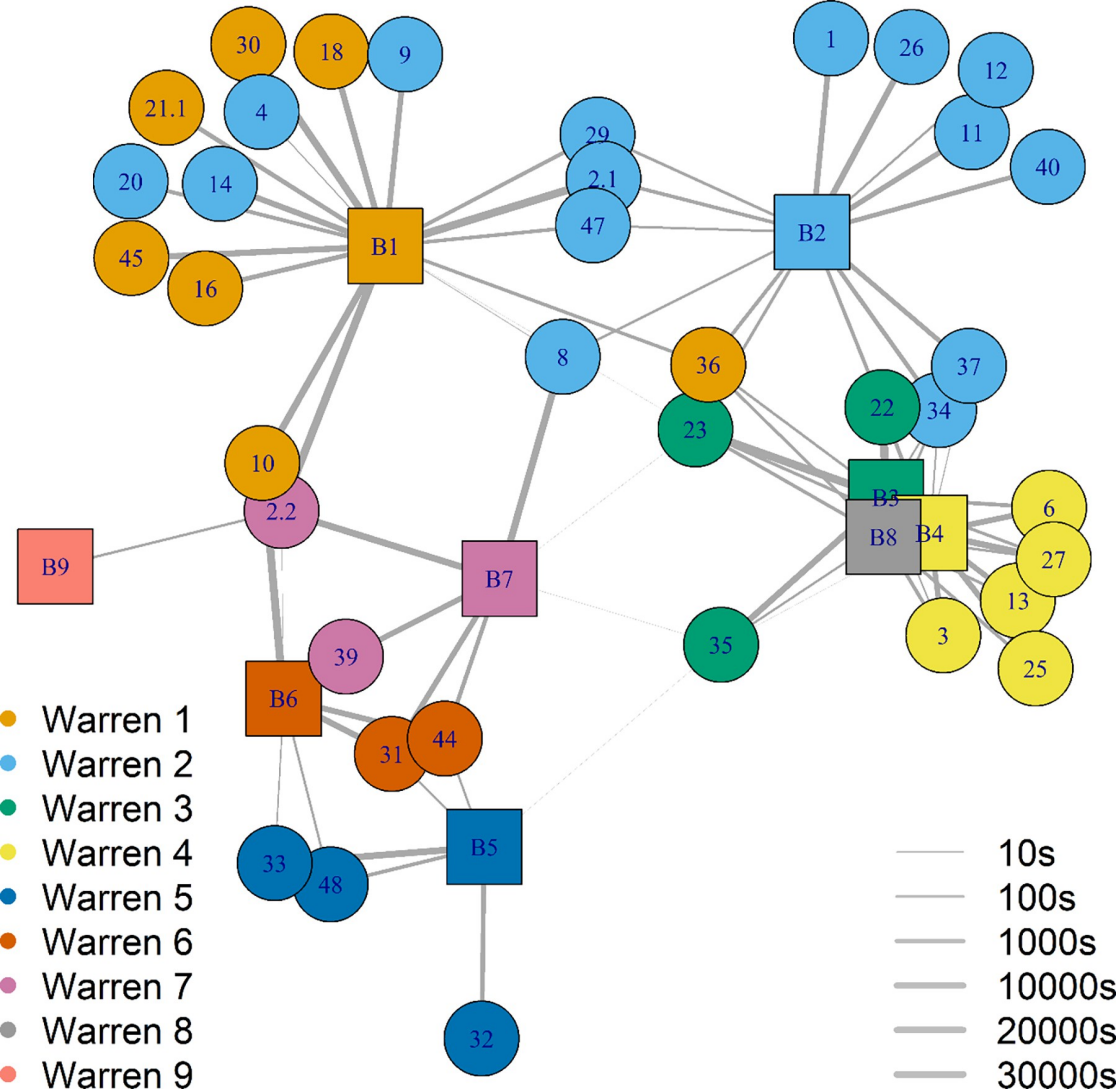
A network of logger pairings with base stations. Nodes (circles indicating logger identification number) represent rabbits and lines between nodes indicate the strength of the pairing. Edge thickness (10–30,000 seconds) is proportional to total time in pairing (log_10_ scale). Nodes and squares are coloured according to the base station location (coloured squares, prefix ‘B’, n = 9) and warren of capture (n = 7) for the loggers. Base stations are numbered as per each warren number.

Of the 12 base stations deployed, two did not produce any data (one each on warren 1 and 2). However, given that these warrens had two base stations deployed, contact data from these warrens were still collected. The base stations logged encounters with 37 rabbits (adult n = 13, juvenile n = 24). Base stations were associated with rabbit’s WOC and are referred to as inter- and intra-warren detections (Fig 5).

**Fig 6:**
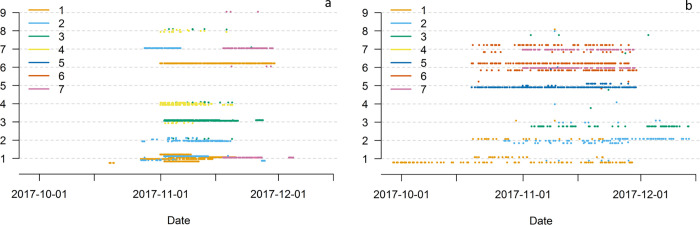
Time stream of juvenile (a) and adult (b) encounters with each base station, represented by warren number (vertical axis). Encounters are colour coded according to warren of capture (n = 7). Each line represents one logger, and vertical positioning of the logger indicates the base station number (warren number, n = 9) of detection. Overlapping data points are scattered for clarity. Only warren 5 had two functioning base stations, and data from both were merged.

Of the 24 juvenile rabbits detected by base stations, 15 visited one or more warren systems (Figs 2 and 5). Most juvenile inter-warren detections were over shorter distances (inter-warren pairing duration Σ = 730,005 sec). For example, the majority of juveniles associated with warrens 3 and 4 were detected at either warrens 3, 4 and/or 9, which were all within 50 m of each other (Figs 1 and 5). Although less frequent, some juveniles did travel greater distances. Two juvenile rabbits (loggers 8 and 23) were detected at four and six different warrens respectively, spanning the 330 m of the study area (Figs 1, 5 and 6A). Interestingly, some juvenile rabbits captured at warren 2 were only detected at warren 1. All but 2 juvenile rabbits (loggers 2.1 and 2.2) had stronger intra-warren connections than adults.

Ten adult rabbits were detected at multiple warrens (2–4 warrens: inter-warren pairing duration Σ = 6,815 sec), three were only detected at their WOC (Figs 5 and 6B) and one adult logger was not detected at all. Males appeared to visit warrens other than their WOC whereas females did not. However, the sample size of these observations was too small for a firm conclusion. The strength of connections varied with some adult rabbits showing strong intra-warren connections (Σ> 1,500 sec, n = 4), while all others displayed similar multiple inter-warren connections. No adults were detected at warren 9, despite it being close to warren 6 and 7 (~35 m). Warren use by adults at warrens 6 and 7 overlapped (loggers 31, 39 and 44: Fig 5), while most other pairings were fleeting in comparison with those of juveniles (Fig 6). Adult logger 32 appeared to have strong connections with warren 5 (WOC), however, excavation determined that it had died in the warren directly below base station 5 (Fig 6B).

## Field tracking detections

**Fig 7:**
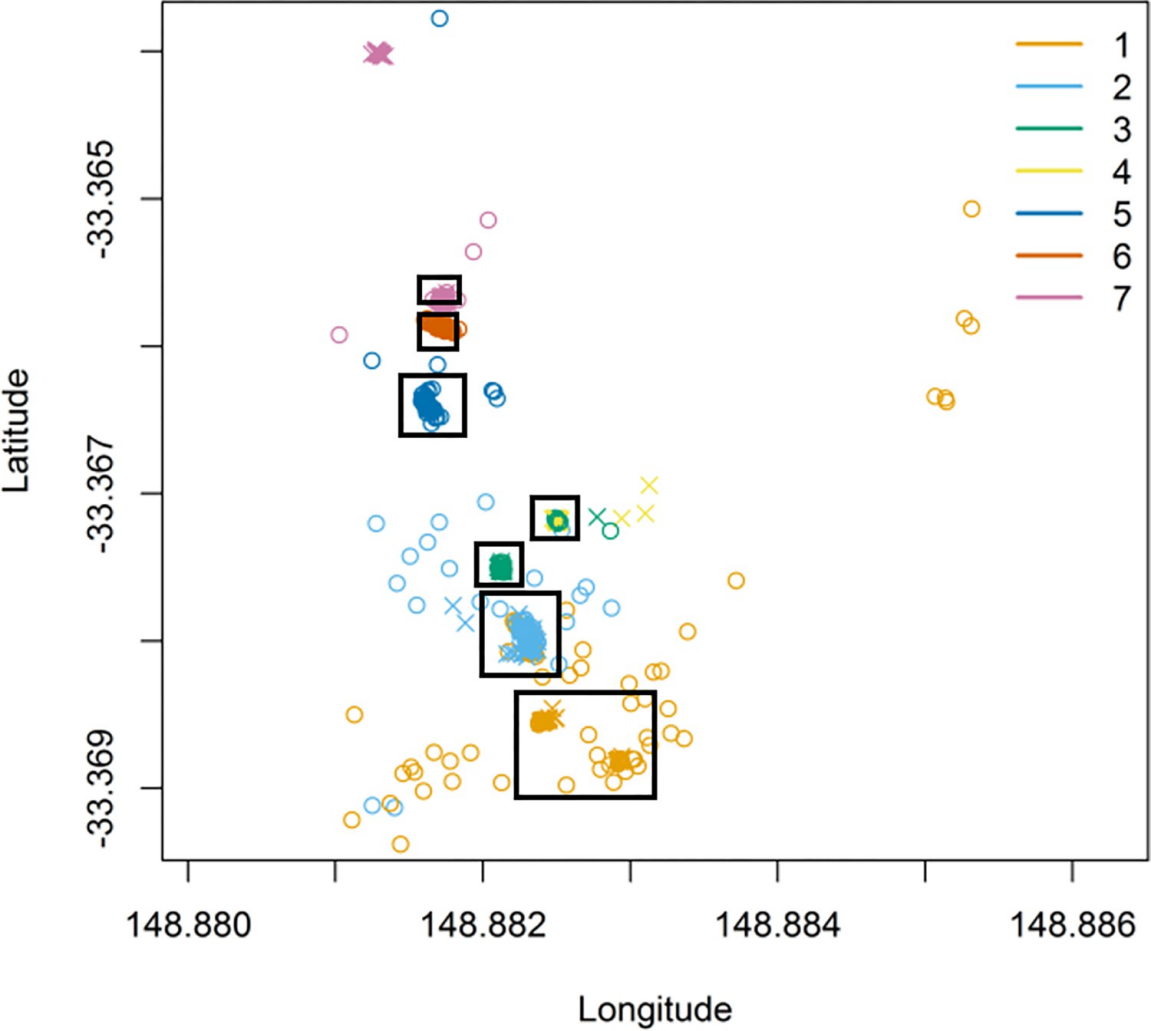
Detections of each logger using a hand-held Yagi antenna and receiver throughout the study. Detections occurred at 05:30, 16:00 and randomly between 22:00–02:00, Monday–Friday. Colours indicate the warren of capture (n = 7). Black squares indicate estimated warren extent. Circles represent juveniles and crosses represent adults. Axes are scaled to equal distance.

Locations collected with the hand-held VHF antenna confirmed that adults used larger areas than juveniles. Although most frequently located in their WOC, six juveniles were occasionally detected in warrens other than their WOC (Fig 7). For example, juveniles from warren 4 were found in warren 3. Similarly, juveniles from warren 1 were found to have moved to warrens at the northern end of the study area. Seven adult rabbits were also detected in multiple warrens during the study (Fig 7). Some adults travelled greater distances than juveniles, for example, adult loggers 34, 36 and 45 and 48 travelled outside of the numbered warrens (greater than 330 m). Four adult rabbits were twice found feeding together away from their WOC during evening surveys; however, pairing between loggers was not recorded.

## Rabbit mortalities

Three rabbits were known to have died during the study. One juvenile rabbit (300 g) and one adult (1000 g) without loggers were confirmed, by an independent laboratory (via serology test), to have seropositive results to GI.2 (approximately halfway through the study period, 8^th^ and 10^th^ of November 2017 respectively), which may have caused their death. The third rabbit (adult logger number 32: 1300 g) tested negative to virus, and the cause of death could also not be determined. We suspected that three other rabbits were taken by predators: one juvenile logger (logger number 21) was found near a red fox (*Vulpes vulpes*) den under a blackberry (*Rubus fruticosus*) shrub and two adult collars (logger numbers 39 and 45) were found with fox bite marks and evidence of chewing. Two other rabbits (logger number 38 and 46) went missing during the study and were not recovered despite searches in and adjacent to the study location.

## Discussion

### Inter-group pairings and population mixing

Our data confirmed that juvenile rabbits could play a substantial role in pathogen transmission through inter-group and inter-warren pairings. Because social networks influence disease networks (at the population level) [31], these inter-group pairings are essential for pathogen transmission in wild populations. This is particularly so for lagoviruses because the main transmission mode is via direct contact [12,21]. Because juvenile rabbits are potentially resistant to RHDV1 and can carry and shed virus [25,47], inter-group pairings with susceptible rabbits from other social groups could be of particular importance in facilitating the transmission of both pathogenic and non-pathogenic RHDV.

Adult-juvenile pairings were more frequent and longer in duration than A–A pairings, and as juveniles can shed virus for many weeks after infection [50], these inter-group pairings potentially enable this cohort to directly and indirectly transmit pathogenic lagoviruses to susceptible adults. As expected, A–A inter-group pairings were very infrequent and short adult rabbit inter-group pairings were infrequent, which corroborates Marsh *et al* [42]. The low frequency of A–A inter-group pairings limits the ability of this age class to exchange virus through direct pairing, which further highlights the potential importance of A–J inter-group pairings in lagovirus transmission.

In our study, J-J inter-group pairings were longer and more numerous than A–J and A–A. These factors are likely sufficient for the expression of behaviours that enable the direct and indirect transmission of virus between social groups. The substantially longer J–J inter-group pairing time increases the likelihood of associated susceptible juveniles becoming infected. The length of time and type of transmission behaviours are also just as important in pathogen exchange within a pathogen network [37]. Transmission behaviours for juvenile rabbits such as cuddling, allogrooming and, in some cases, aggression, promote the likelihood of bodily fluid (saliva, urine, faecal matter and blood) exchange [54]. These behaviours, often longer in duration, can facilitate pathogen transmission [10,54]. The duration of inter-group J–J pairings we observed suggest there was ample opportunity for transmission behaviours to occur, resulting in the potential transmission of lagoviruses. However, we do not know the exact type of behaviour that occurred during the pairings, which could further influence the likelihood of lagovirus transmission.

Due to particularly high connectivity, some individuals could play a larger role in lagovirus transmission than others. Highly connected individuals, or ‘superspreaders’ [55,56], have a greater capacity to transmit virus at a population level, particularly in species such as rabbits, as these individuals form connections between social groups that might not occur between adults [25,30,35]. Almost all juvenile rabbits paired with juveniles from neighbouring social groups, and some with potentially susceptible neighbouring adults. Given J–J and A–J pairings were longer and more numerous than A–A, some juvenile rabbits could be potential superspreaders of lagoviruses because they were well-connected over multiple warrens and social groups.

Juvenile rabbits are likely also important for spreading acquired immunity. Like RHDV, RHDV GI.4c can be shed by juveniles up to seven days after infection, providing temporary immunity against RHDV1 [22]. This further complicates rabbit disease networks, as the spread of RHDV GI.4c can create a resistant breeding population [25,26]. Through their strong connections, juveniles therefore have a greater ability to transmit RHDV GI.4c, which could increase immunity against RHDV1 in rabbit populations.

## Intra-group pairings

Pairings between members of the same group are also important for pathogen transmission [10,35,47]. Juvenile rabbits had the strongest intra-group connections. The high connectivity of juvenile rabbits with littermates and their mothers during the early weeks of life that we observed has been shown in other studies [2,54]. The likelihood of between-juvenile rabbit pairings within the warren is inherently higher because, given that the average litter size is four-six kittens per female [1] and that a female produces between 18 and 30 young per year [2], juveniles are more common than adults during breeding seasons. This likely increases the probability of virus transmission between littermates and to susceptible individuals within their group. As RHDV typically takes one-two days to kill its host [6], and juveniles have heightened protection from lethal lagoviruses due to maternal antibodies (up to approximately 12 weeks) [27], this well-connected cohort has an increased likelihood of encountering multiple susceptible individuals during this time.

Juvenile rabbits are naturally resistant to RHDV1s up to about six weeks of age [45], but resistant individuals can shed a lethal virus load post-infection for up to 10 weeks [25]. The strength of juvenile intra-group connections means this cohort has the potential to transmit virus to almost all members of their social group. Greater virus spread within groups increases the likelihood of virus spread between groups. This increases the likelihood of individuals spreading infection and/or immunity across populations.

## Multiple warren use

More than half of the juvenile rabbits used multiple warrens, confirming the potential of them spreading and shedding virus outside of their WOC. Juvenile rabbits can travel up to one and a half kilometres from their natal site [2,33]. This is consistent with our finding that juvenile rabbits moved across the entire study area (approximately six hectares) and encountered potentially susceptible adults from different social groups. This is particularly important for pathogen transmission because juveniles are capable of transmitting virus to neighbouring warrens and over great distances. Furthermore, because the exploration range of juvenile rabbits increases as they grow and age [57], the pathogen transmission range could follow a similar trend. This highlights the critical role that juveniles can play in facilitating disease epidemics in this system. Further investigation into the relationships between age, distance travelled, and the number of inter-group and inter-warren pairings may help define an age threshold for maximum mortality by infection.

Exploration by juvenile rabbits prior to dispersal provides them with information on resource availability and hierarchical structure of neighbouring groups [2,32,48]. Although most juveniles spent most of their time at their WOC, there were four cases where juveniles were only ever detected away from their WOC. It was possible that these juveniles were trapped during exploratory events, and therefore their WOC was not their natal warren. Another explanation is that they were never detected by the base station on their natal warren and were only ever detected during their exploratory events on other warrens. Given the large size of these warrens (warrens 1 and 2), this second option is possible.

Not all individuals within a warren system use or have access to the entire warren [23]. Although juvenile rabbits are likely to have less restriction moving through their natal warren, our observed dispersion by juvenile rabbits suggests that most exploration is likely to be out of the natal warren rather than within. These findings raise three important points for pathogen transmission: 1) juvenile rabbits might not interact with all surrounding social groups; 2) as juveniles prepare to disperse, they spend more time investigating warrens and social groups away from their natal warren; and 3) juvenile exploration could partially account for pulsing of disease outbreaks because births often pulse in response to seasonal conditions [40], as did dispersals and interactions between warrens and warren clusters. This also implies that flow rate for pathogen transferred by juvenile rabbits to other social groups will vary because some groups will not be socially connected. Under these circumstances, transmission of pathogens would depend on other modes such as insect vectors [13].

Surprisingly, most adult rabbits were detected by base stations at more than one warren. Previous studies using VHF tracking found that most adult rabbits remained within the confines of their social territories [42]. However, it is likely that infrequent instances of pairing with other warrens were not detected during that study because of limitations of VHF tracking. The majority of such warren visits were by rabbits in close proximity to their WOC, and as a whole, warren visits were largely infrequent and short. Our data indicated that male adults were more likely to visit warrens other than their WOC. This is consistent with the finding of DiVincenti and Rehrig [48] that female rabbit home ranges were often smaller, while male home ranges were larger and influenced by availability of breeding females. Therefore, use of multiple warrens by adults in our study was likely driven by demographic factors such as sex and reproductive status, particularly as the study was undertaken during the late breeding season. The timing of inter-warren visits is likely to influence pathogen transmission. If adults are covering greater distances during particular times of the year (e.g. breeding seasons [25]), the chance of lagovirus transmission through both direct and indirect pairing with juveniles from multiple warrens is increased.

## Effects of mortality

Several mortalities were observed in our study, and both GI.2 and predation were identified as the causes of the mortality events. The outbreak of GI.2 could have affected the pairing frequency and duration of pairings. We possibly underestimated the frequency of pairings between rabbits, and potentially overestimated the duration of pairings, e.g. the 5 hr 51 min outlier pairing between an adult and a juvenile (Fig 2) could have been between live and moribund infected rabbits.

Because rabbits form a significant part of introduced predator diets (wild dogs [58], foxes and cats (*Felis catus*) [39]), predation or scavenging of infected carcases can aid in transmission of pathogens through faecal shedding by predators over their home range [10]. Furthermore, transmission of lagoviruses could be facilitated by insects around areas of high predation activity (e.g. fox dens) due to greater presence of dead infected rabbits [13]. Although we observed mortalities that were likely due to predation, these were too sparse to inform us about the likely impacts on pathogen transmission.

## Further considerations

It is unknown what effects that lagovirus observed clinical signs [9,34,43,59], population size, dispersion and density, and landscape features (e.g. topography, soil structure) had on rabbits’ behaviours and movements, and which could therefore influence the spread of lagoviruses. Our study population was localised but of high density for Tablelands regions (1–16 rabbits ha^-1^ [12]) and might not have been representative of contact networks in other environments. Further research into the effects of lagovirus infection and different rabbit population sizes on animal movement and transmission behaviours is suggested. In addition, it is possible that the rabbits trapped and monitored during our study exhibited different behaviours to those that were not trapped and monitored.

## Conclusions

As lagoviruses are spread by both direct and indirect pairing, the high connectivity of juvenile rabbits may increase their potential in transmitting the pathogens across social groups. In particular, the strength of A–J inter-group pairings and juvenile inter-warren use, confirmed the potential for juveniles to act as superspreaders of lagoviruses [37,55]. This is of particular importance for RHDV-mediated biocontrol transmission, as juvenile rabbits can shed RHDV for many weeks following infection, without developing the disease. This has implications for the timing of virus release, and further investigation should focus on the effect this may have on a resistant breeding population versus increased virus spread for the management of wild European rabbits [24]. Further investigation should also be made into the effect of juvenile shedding status, and temporal variation in juvenile rabbit behaviour on the transmission of lagoviruses could lead to improved timing of RHDV-mediated biocontrol and better management of lagoviruses for the preservation of rabbit populations in their native range [11,60].

## Supporting information

S1 Table Compiled logger data. (XLSX)

S2 Table EBA -D. Carlise-may-Aug-2020 excel conver.
(XLSX)

S3 Table Honours capture history. (XLSX)

S4 Table Logger vs warren. (XLSX)
